# Internet-delivered therapy for alcohol misuse: engagement, satisfaction, and outcomes when patients select their preference for therapist- or self-guided treatment

**DOI:** 10.1186/s13722-024-00456-8

**Published:** 2024-04-20

**Authors:** Ram P. Sapkota, Tristen Lozinski, Andrew Wilhems, Marcie Nugent, Michael P. Schaub, Matthew T. Keough, Christopher Sundström, Heather D. Hadjistavropoulos

**Affiliations:** 1 Online Therapy Unit, Department of Psychology, University of Regina, 3737 Wascana Parkway, Regina, SK S4S 0A2 Canada; 2 Swiss Research Institute for Public Health and Addiction, University of Zurich, Konradstrasse 32, 8005 Zurich, Switzerland; 3 Department of Psychology, York University, 4700 Keele Street, Toronto, ON M3J 1P3 Canada; 4 Department of Psychology, Stockholm University, 106 91 Stockholm, Sweden; 5 Department of Clinical Neuroscience, Centre for Psychiatric Research, Karolinska Institutet, Norra Stationsgatan 69, 113 64 Stockholm, Sweden

**Keywords:** Alcohol, Substance use, Treatment, Internet-delivered cognitive behaviour therapy, Preference trial

## Abstract

**Background:**

Alcohol misuse is common and causes substantial harm. Internet-delivered cognitive behaviour therapy (ICBT) is effective in reducing alcohol misuse; however, the literature investigating how treatment outcomes are impacted by patients’ preferences for therapist- versus self-guided ICBT for alcohol misuse is sparse.

**Methods:**

In this preference trial, 74 eligible patients (who reported ≥ 14 drinks in the previous week and obtained scores suggesting hazardous or harmful drinking) chose between enrolling in either therapist- or self-guided ICBT for alcohol misuse. We investigated whether those who chose therapist- versus self-guided ICBT differed in their (a) drinking outcomes—as measured by Timeline Follow-Back (TLFB) and heavy drinking days (HDD) at post-treatment and 3-month follow-up—and (b) post-treatment ICBT engagement and satisfaction.

**Results:**

The majority (81.1%) of eligible patients chose therapist-guided ICBT. These patients reported higher psychotropic medication use, drinking difficulties, and anxiety symptoms. For both the therapist- and self-guided patients, a modified intention-to-treat analysis revealed large within-group treatment effects for TLFB (β =  − 2.64, SE 0.66; p < 0.001) and HDD (β =  − 0.34, SE 0.07; p < 0.001), with large pre-to-post-treatment Cohen’s effect sizes of d = 0.97 (95% CI [0.49, 1.45]) for TLFB and d = 1.19 (95% CI [0.69, 1.68]) for HDD. The interaction comparing the effects of therapist- to self-guided ICBT over time was not significant for TLFB (p = 0.34) or HDD (p = 0.06). With treatment, for both therapist- and self-guided patients, there was a significant improvement in drinking difficulties, cravings, and confidence with controlling cravings, as well as in anxiety, depression, and functional impairment. Further, the majority (75.7%) of patients completed five or more lessons, as well as reported overall satisfaction with the treatment (88.9%) and increased confidence in managing their symptoms (86.7%); these outcomes also did not differ between therapist- and self-guided patients.

**Conclusions:**

The current study shows that ICBT for alcohol misuse is associated with reduced drinking and comorbid mental health difficulties over time, irrespective of whether patients chose to complete the course on their own or with therapist guidance.

*Trial registration number:* NCT04611854 (https://clinicaltrials.gov/ct2/show/NCT04611854).

## Background

Alcohol misuse is one of the leading contributors to morbidity and mortality worldwide. Alcohol misuse results in substantial health care costs and lost work productivity in addition to the psychological, interpersonal, and chronic health difficulties associated with high levels of alcohol use [1–3]. Unfortunately, only a small fraction of individuals struggling with alcohol misuse seek and/or receive treatment, often due to barriers such as stigma, lack of available services in primary care locations, or low perceived helpfulness of accessible treatments [4–6]. There is growing acknowledgement of Internet-delivered cognitive behaviour therapy (ICBT) as an innovative and evidence-based treatment method for overcoming some of these barriers to seeking and receiving face-to-face alcohol misuse treatment [7].

Systematic reviews and meta-analyses have found Internet interventions (i.e., brief interventions and ICBT) to be effective in reducing alcohol misuse [7–9]. There are inconsistent findings, however, regarding the added benefits of therapist guidance on treatment outcomes. For instance, while the largest meta-analysis published to date [i.e., 9] found significant differences in favour of therapist guidance, several recent studies (e.g., [10–13]) have failed to find such differences. One factor that has not been given much consideration with respect to therapist guidance in ICBT for alcohol misuse is how patients’ preferences for therapist- versus self-guided treatment impacts enrollment, engagement, satisfaction, and outcomes.

According to a review by the American Psychological Association’s Presidential Task Force on Evidence-Based Practice [14], patients’ preferences significantly influence study enrollment and attrition, as well as treatment adherence, satisfaction, and outcomes [15]. When enrolled in their non-preferred treatment, patients may experience *resentful demoralization*, where they become less motivated to adhere to treatment, thereby negatively impacting treatment outcomes [16].

Review of the literature on ICBT for alcohol misuse has identified only one study that considered patients’ preferences, but that study did not examine preference for self-guided versus therapist-guided ICBT. Rather, in that study [17], participants were randomized to guided or unguided ICBT. Among those who were randomized to receive guidance, participants in one group selected between asynchronous (i.e., messages) versus synchronous messages (i.e., chat) from therapists, while the other group received asynchronous messages only. The results showed that 65% of the participants given a choice preferred asynchronous messages. Regardless of their choice, both guidance groups reported significantly lower past week alcohol consumption and drinking difficulties compared to the group without guidance.

Given that there has been limited research on patients’ preferences in ICBT for alcohol misuse [17], we aimed to address this prominent research gap by elucidating patients’ preferences for therapist- versus self-guided ICBT. While some evidence suggests that therapist-guided Internet-delivered interventions are more effective than self-guided interventions [9], various studies have found similar benefits with therapist- and self-guided approaches [10–13]. In addition to these unclear findings, it is also not known what patients prefer, what characteristics differentiate between those who prefer self-guided versus therapist-guided treatment, or how their preferences may impact treatment engagement, satisfaction, and outcomes.

In this study, we examined treatment preferences and then whether patients in their preferred condition, namely therapist- versus self-guided ICBT differed in their drinking outcomes, as well as in their treatment engagement and satisfaction. Based on past research [7, 9, 10, 12, 13], we predicted that—for both those who chose to enroll in therapist- and self-guided ICBT—patients would improve their drinking outcomes with treatment. Further, because these patients chose their guidance level, we expected to see similar treatment engagement and satisfaction between those in therapist- and self-guided ICBT; however, acknowledging the evidence supporting enhanced engagement alongside therapist guidance [12], we expected that we might see slightly higher engagement among patients who chose therapist-guided ICBT.

In separate exploratory analyses, we examined the treatment engagement, satisfaction, and outcomes of self-guided ICBT for alcohol misuse among patients who were not eligible to receive therapist guidance because they did not reside in Saskatchewan (SK; the province funding the therapists and where our therapists were registered to practice) and were therefore automatically assigned to self-guided ICBT. This exploratory research allowed for an interesting natural comparison to the SK-residing patients who were eligible to select their treatment preference. It was particularly interesting to explore if those who received their preference for self-guided ICBT would show greater engagement, satisfaction and outcomes than those who were automatically enrolled in the self-guided ICBT.

## Methods

### Trial design

In the current preference trial, patients completed an 8-week ICBT program for alcohol misuse, named the Alcohol Change Course (ACC). After a brief screening interview, eligible patients were given a choice between (a) a therapist-guided version, where patients could message an assigned therapist who responded on a pre-determined day each week or (b) a self-guided version, where patients worked on the program on their own. The trial was conducted via the Online Therapy Unit (OTU) website (www.onlinetherapyuser.ca). The OTU is based at the University of Regina, SK, Canada, offering free therapist-guided ICBT to Saskatchewan residents with funding awarded by the Saskatchewan Ministry of Health. In addition, the self-guided ACC is available to all Canadian residents. The current study was registered at https://clinicaltrials.gov/ct2/show/NCT04611854 (NCT04611854) and approved by the University of Regina Ethics Review Board (approval number 2019-058). All patients signed an online informed consent form before enrolling in the study.

### Patients

Patients were recruited through several sources, including online advertisements, emails, posters distributed to SK physicians/doctors, referrals from SK health regions, and word of mouth from friends and family members. These referral sources directed interested individuals to the OTU website, where they could read about the ACC and apply to take the course through an online screening questionnaire. The questionnaire included a consent form and questions regarding applicants’ contact information (e.g., telephone number, email address), demographic information (e.g., gender, ethnicity, education), relevant personal details (e.g., medical history), mental health (e.g., depression, anxiety), and alcohol use. Upon completing the screening, applicants meeting initial inclusion criteria were directed to an online booking program to schedule a telephone screening appointment with OTU staff. During the screening call, applicants answered follow-up questions to confirm their eligibility. Patients eligible for the preference trial had to: (a) be ≥ 18 years old; (b) be a SK resident; (c) have Internet access; (d) have consumed ≥ 14 drinks in the previous week (i.e., a cut-off used in similar previous research; i.e., [13]); and (e) indicate hazardous or harmful alcohol consumption by scoring ≥ 8 on the Alcohol Use Disorder Identification Test [18]. Additionally, applicants were excluded if they (a) scored ≥ 25 on the Drug Use Disorder Identification Test [DUDIT; [19]]; (b) scored a three on item 9—asking about suicidal ideation—of the 9-item Patient Health Questionnaire [PHQ-9; [20]]; (c) had unmanaged symptoms of bipolar disorder, schizophrenia, and/or psychosis; (d) had a medical condition that would inhibit active participation in the course; (e) received concurrent mental health treatment more than twice monthly in the last three months (not including taking psychotropic medication[s]); or (f) had been hospitalized for mental health reasons in the past year.

Patients that met all of the listed criteria were eligible to choose between the self-guided and therapist-guided ACC and were subsequently analyzed in the current trial. Additionally, those patients who met all of the criteria, apart from residing in SK, were offered self-guided ACC and were included in the exploratory analyses of the current study.

### Intervention: the alcohol change course

The ACC is an ICBT program for alcohol misuse, developed initially in Switzerland [21–23]. The intervention was translated to English [24] and subsequently adapted to address young adults’ alcohol use and depression [24, 25]. As described previously [12], the ACC was further modified for use in the OTU, being revised to focus on adult populations and to align with the other evidence-based ICBT programs offered by the OTU. The updated version of the ACC contained 12 lessons—delivered consecutively over eight weeks—comprised of slide shows with psychoeducation, case stories, and downloadable worksheets for practicing skills. For the current preference trial, a patient-oriented working group (i.e., three patients with personal or familial lived experience with alcohol misuse, two Internet therapists, two operations managers, two trainees, and two group facilitators) collaborated in updating the ACC based on patient feedback, altering the language to be more inclusive and condensing the course into eight lessons, one lesson per week [26]. Furthermore, information regarding abstinence, the relationship between physical health and drinking, as well as Canada’s alcohol use prevalence rates and low-risk drinking guidelines was incorporated into the introductory lesson.

### Treatment guidance

Eligible patients were given a choice between the therapist-guided and self-guided versions of the ACC. Regardless of patients’ preferred treatment condition, they received automated, weekly emails containing information regarding new lesson content, and they were also contacted by the OTU if they wanted to discontinue the ACC and/or experienced technical difficulties with the treatment platform. Moreover, patients in both the therapist- and self-guided ACC responded to weekly surveys prompting them to reflect on their challenges and learning experiences during the past week.

Apart from these specific situations, patients who chose the self-guided ACC received no regular contact with Internet therapists. In contrast, patients who chose the therapist-guided ACC were supported by one of two Internet therapists; these therapists held graduate degrees as a Master of social work and a Master of Education in counselling psychology, and had practiced for 14 and two years, respectively. Through the messaging functioning of the online treatment platform, therapists contacted patients once per week on a pre-set day, spending ~ 15 weekly minutes connecting with each therapist-guided patient to help them manage motivation, reinforce lesson completion, and answer questions. Therapists also communicated with these patients via telephone calls in rare cases where (a) patients demonstrated increased suicide risk, (b) patients requested a call, and/or (c) a misunderstanding between therapists and patients was to be addressed.

### Instruments

#### Primary outcome measures

At pre-treatment (baseline screening), mid-treatment (Week 4), post-treatment (Week 8), and follow-up (Week 20), total preceding week alcohol consumption and number of heavy drinking days (HDD) were assessed using the well-standardized Timeline Follow-Back [TLFB; [27]]. Participants reported the number of standard drinks (i.e., one 12 oz can/bottle of 5% beer, cider, or alcopop/cooler; one 4.5 oz glass of 12% wine; or one 1.3–1.5 oz shot of 40% hard liquor) that they had consumed during the past seven days at each measurement period. The seven daily values were summed to calculate the number of total preceding week drinks (i.e., the TLFB variable). Cronbach’s α for the TLFB ranged from 0.74 to 0.82 in the current study. Further, separated by gender, the number of days when women consumed more than three daily drinks and when men consumed more than four daily drinks in the past seven days were summed to calculate their total number of preceding week HDDs.

#### Secondary outcome measures

All secondary outcome measures were assessed at pre-treatment, post-treatment, and follow-up using well-standardized self-report questionnaires.

Alcohol misuse was assessed through the Alcohol Use Disorder Identification Test [AUDIT; [18]]. Patients responded to 10 items on scales from 0 to 4; responses were summed to produce a total score from 0 to 40. Higher scores indicate greater alcohol-related difficulties and behaviours. Scores ≥ 8 indicate hazardous or harmful alcohol consumption, and scores ≥ 15 indicate a possible alcohol use disorder. In the current study, Cronbach’s α for the AUDIT ranged from 0.77 to 0.83.

Alcohol craving was assessed via the Penn Alcohol Craving Scale [PACS; [28]]. Patients responded to five items on 7-point scales from 0 to 6. Items were summed to produce a total score ranging from 0 to 30, with higher scores indicating greater alcohol craving. Cronbach’s α for the PACS ranged from 0.91 to 0.92 in the current study.

Patients’ confidence in their ability to resist alcohol cravings was assessed by the Brief Situational Confidence Questionnaire [BSCQ; [29]]. Patients responded to items referencing eight situations (i.e., negative emotional states, negative physical states, positive emotional states, testing personal control, urges and temptations, interpersonal conflict, social pressure, and positive social states), each measured on a scale from 0 to 100. Item responses were summed to produce a total score from 0 to 800. Higher scores indicate greater confidence in one’s abilities to resist alcohol cravings. In the current study, Cronbach’s α for the BSCQ ranged from 0.87 to 0.91.

Anxiety symptoms were assessed with the Generalized Anxiety Disorder-7 [GAD-7; [30]]. Patients responded to seven items on 4-point scales from 0 to 3. Responses were summed to produce a total score, ranging from 0 to 21. Higher scores indicate more severe self-reported anxiety, with scores > 9 indicating clinical levels of anxiety. Cronbach’s α for the GAD-7 ranged from 0.89 to 0.90 in the current study.

Depression symptoms were assessed via the 9-item Patient Health Questionnaire [PHQ-9; 20]. Patients responded to nine items on 4-point scales from 0 to 3. Items were summed to produce a total score from 0 to 27. Higher scores indicate greater depression symptom severity, with scores > 9 indicating a possible major depressive disorder. In the current study, Cronbach’s α for the PHQ-9 ranged from 0.87 to 0.91.

Functional impairment was assessed with three items from the Sheehan Disability Scale [SDS; [31]]. Patients responded to three items (on 11-point scales from 0 to 10) assessing work/school, social, and family life functional impairment. Responses were summed to yield a total score ranging from 0 to 30, where higher scores indicate greater total functional impairment. Cronbach’s α for the SDS ranged from 0.82 to 0.93 in the current study.

#### Additional measures

At baseline screening, patients completed the Drug Use Disorder Identification Test [DUDIT; [19]], an 11-item self-report scale with total scores ranging from 0 to 44. The first nine items are rated 0, 1, 2, 3, and 4, while the last two items are rated 0, 2, and 4. A score of ≥ 25 suggests significant problem with drugs and was used to exclude patients from the current trial. The Cronbach’s α was 0.74 for the DUDIT in the current trial.

At baseline screening, patients’ motivation to change their drinking and current stage of change (i.e., pre-contemplation, contemplation, action) were assessed by the revised version of the self-report Readiness to Change Questionnaire—Treatment Version [RCQ-TV; [33]]. Twelve items were scored on a 5-point scale ranging from − 2 = *strongly disagree* to 2 = *strongly agree*. Item responses were used to calculate three sum scores ranging from − 8 to 8 for pre-contemplation (Cronbach’s α = 0.83), contemplation (Cronbach’s α = 0.70), and action (Cronbach’s α = 0.92). Patients were considered to be in the stage of change where they received their highest sum score. See Heather and Hönekopp [33] for further information regarding the revised RCQ-TV scoring.

At mid-treatment, patients’ perceived treatment credibility and expected treatment success were assessed via the 6-item Credibility and Expectancy Questionnaire [CEQ; [32]]. Patients responded to the first three items, each on a 9-point scale ranging from 1 = *not at all logical/useful/confident* to 9 = *very logical/useful/confident*; these items were summed to produce a total score ranging from 3 to 27, with higher scores indicating greater perceived treatment credibility. Further, patients responded to the last three items; items 4 and 6 were on 11-point scales ranging from 0 to 100 (coded as 0–10), and item 5 was on a 9-point scale ranging from 1 to 9. These three items were summed to produce a total score ranging from 1 to 29, with higher scores indicating greater treatment expectancy. Cronbach’s α = 0.80 for the CEQ in the current study.

At post-treatment, patients’ satisfaction with and negative effects experienced during the ACC were assessed via a self-report questionnaire developed by the OTU research team [see [26]]. Patients responded to 10 items measuring their evaluations of the treatment (e.g., “Would you feel confident recommending this treatment to a friend?”) and negative effects they perceived experiencing as a result of the treatment (e.g., “Have you experienced any unwanted negative effects or events that you associate with taking part in this online treatment?”).

Further, as a proxy for engagement, patients’ course completion was recorded throughout their eight weeks of treatment. Total values representing the proportion of completed lessons and the proportion of overall treatment completion were calculated for each patient.

### Statistical analyses

Statistical analyses were conducted using IBM SPSS Statistics (Version 27.0). Descriptive statistics described patients’ characteristics in percentages, means, and standard deviations. The pre-treatment characteristics and post-treatment engagement and satisfaction of the groups (i.e., therapist- versus self-guided) were compared with Chi square tests for categorical variables and *t* tests for continuous variables. Following previously established methodology [34–36], a series of mixed model analyses were conducted to examine changes in the primary (i.e., TLFB, HDD) and secondary (i.e., AUDIT, PACS, BSCQ, GAD-7, PHQ-9, SDS) outcomes over time and to assess if these changes differed between the groups. Of note, this approach was chosen as mixed-model analyses can produce accurate inferences with small samples [see 41, 42]. For each outcome, a series of models involving the fixed and random effects of intercept (i.e., symptom scores at pre-treatment) and slope (*time*) were conducted and included in the model to account for the correlated nature of the data. The fixed-effect models included *time*, the preference group (*group*), and their interaction (*time* × *group*). Intraclass correlation coefficients were manually calculated to determine if mixed model analyses were appropriate [34]. Various within-individual covariance structures (e.g., scaled identity, diagonal, autoregressive, unstructured) were also tested. The models with smallest Akaike’s Information Criterion and Bayesian Information Criterion were selected for the final analysis. Estimates were calculated using the full information maximum likelihood method with the Satterthwaite approximation for the denominator’s degrees of freedom. When significant differences emerged between the preference groups, baseline demographic characteristics were included in the model as covariates to adjust for potentially confounding effects of these variables on treatment outcomes. We also controlled for the potential effects of other clinically relevant independent variables such as concomitant use of psychotropic medications, number of lessons completed (weighted by the actual number of lessons completed), treatment expectancy and credibility, and readiness to change (i.e., pre-contemplation, contemplation, action) by covarying the variables in each of the final models.

Via a series of mixed-models, an exploratory analysis assessed the change in outcomes among the patients who resided outside of SK and, therefore, were automatically assigned to the self-guided ACC as they were not eligible to select their treatment preference. Further, using Chi square tests or *t* tests, post-treatment engagement and satisfaction were compared between the self-guided patients residing outside of SK and the preference trial groups.

#### Missing data management

For the preference trial patients, there were no missing values across all pre-treatment variables but there were 17 (23.0%) and up to 29 (39.2%) patients with missing values in the primary and secondary outcome measures, respectively, at post-treatment. Data were missing from 30 (40.5%) patients at follow-up. There were no significant differences in attrition between therapist- and self-guided preference groups at mid-treatment, post-treatment, and follow-up for the primary outcome variables (*p* range: 0.11–0.85). The analysis of missingness with Little’s Missing Completely at Random test (χ^2^ = 199.86, df = 206, p = 0.61) suggested that the data were missing at random [37]. Following a modified intention-to-treat approach, the missing data were imputed using the multiple imputation method, generating 10 multiply imputed datasets so that the data from all eligible preference trial patients were analyzed [38]. Pooled results are presented for all mixed model analyses. Effect sizes, Cohen’s *d* [39]—for the difference between pre- and post-treatment and pre-treatment and follow-up assessments on the TLFB and HDD variables—were computed using estimated marginal means.

## Results

### Baseline sample and characteristics

**Fig. 1:**
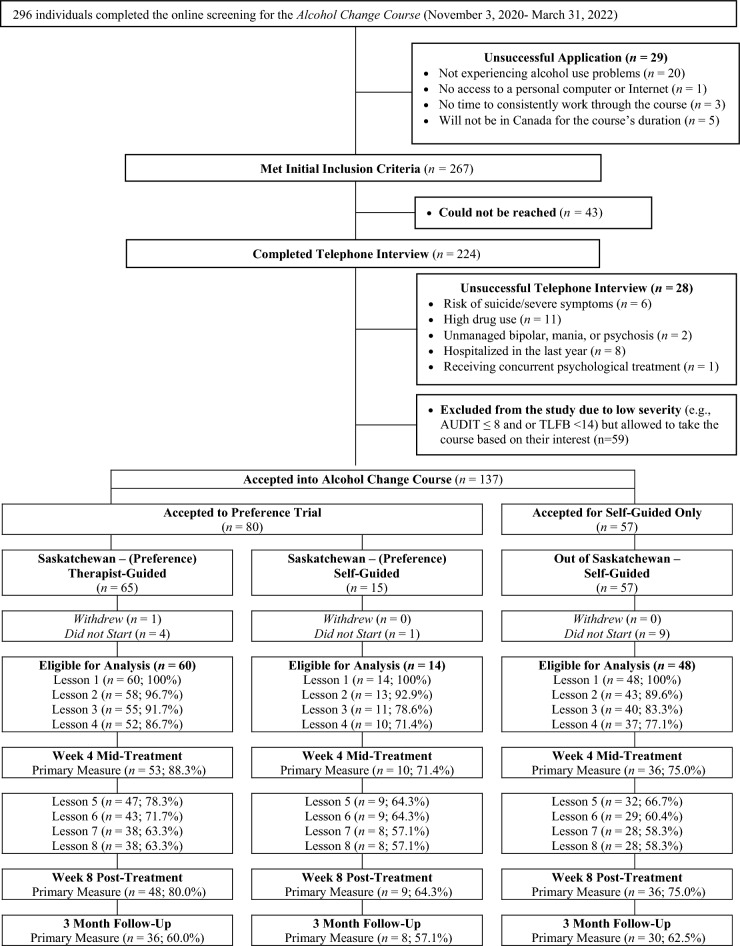
Flow chart

Between November 2020 and March 2022, 80 patients from SK met inclusion criteria for the ACC and selected their preference for the therapist-guided or self-guided ACC (see Fig. 1 for details). Of these patients, one formally withdrew and five did not start treatment, leaving 60 patients who selected the therapist-guided ACC and 14 who selected the self-guided ACC. As part of the exploratory analyses, there were 57 patients who were eligible for the ACC, but resided outside of SK and thus were assigned to the self-guided ACC; 48 of these patients started the ACC.

The descriptive statistics for ACC patients’ demographic and clinical characteristics are detailed in Table 1. The 74 patients who were eligible to choose between the therapist- or self-guided ACC, were primarily in middle adulthood (M = 45.61, SD = 13.29, range = 22–76), women (59.5%), Caucasian (87.8%), married (59.5%), educated beyond high school (79.7%), employed part- or full-time (68.9%), and located in a large city (48.6%). There were no significant demographic differences between those in therapist-guided (n = 60) and self-guided (n = 14) ACC. However, there were several significant group differences in both the secondary outcome and other clinically relevant variables. Significantly more patients in the therapist-guided ACC reported taking psychotropic medication than those in the self-guided ACC. Similarly, although the mean AUDIT scores in both groups suggested a possible alcohol use disorder, patients in the therapist-guided ACC reported significantly higher scores than did those in the self-guided ACC. Anxiety symptoms also significantly differed between the two groups, with patients in the therapist-guided ACC reporting higher anxiety symptoms. Lastly, the therapist- and self-guided groups significantly differed in their current readiness to change their drinking habits, such that patients in the self-guided ACC reported higher pre-contemplation than did patients in the therapist-guided ACC. There were no additional significant differences in baseline characteristics among the preference trial patients enrolled in the therapist- versus self-guided ACC.

**Table 1:** Pre-treatment demographic and clinical characteristics by treatment group

Variable		All patients (*n* = 122)	All Preference Trial patients (*n* = 74)	Preference Trial (*n* = 74)		Accepted for Self-Guided Only (*n* = 48)	Statistical Significance^a^ *χ*^2^ or *t*-test
				Guided (*n* = 60)	Self-Guided (*n* = 14)	Met Severity criteria but Out-of-Province (*n* = 48)	
		*n* (%)	*n* (%)	*n* (%)	*n* (%)	*n* (%)	
Participant Pre-Treatment Characteristics							
Age							
	Mean (*SD*)	45.27 (13.39)	45.61 (13.29)	44.80 (13.09)	49.07 (14.06)	44.75 (13.67)	*p* = .28
	Range	20–76	22–76	22–72	30–76	20–67	
Gender							
	Men	47 (38.5)	29 (39.2)	21 (35.0)	8 (57.1)	18 (37.5)	*p* = .14^b^
	Women	74 (60.7)	44 (59.5)	38 (63.3)	6 (42.9)	30 (62.5)	
	Non-binary	1 (0.8)	1 (1.4)	1 (1.7)	0 (0.0)	0 (0.0)	
Marital status							
	Single/never married	25 (20.5)	18 (24.3)	16 (26.7)	2 (14.3)	7 (14.6)	*p* = .59
	Married/common law	77 (63.1)	44 (59.5)	35 (58.3)	9 (64.3)	33 (68.8)	
	Separated/divorced/widowed	20 (16.4)	12 (16.2)	9 (15.0)	3 (21.4)	8 (16.7)	
Education							
	High school diploma or less	25 (20.5)	15 (20.3)	10 (16.7)	5 (35.7)	10 (20.8)	*p* = .28
	Post high school certificate/diploma	47 (38.5)	31 (41.9)	26 (43.3)	5 (35.7)	16 (33.3)	
	University education	50 (41.0)	28 (37.8)	24 (40.0)	4 (28.6)	22 (45.8)	
Employment status							
	Employed part-time/full time	88 (72.1)	51 (68.9)	44 (73.3)	7 (50.0)	37 (77.1)	*p* = .09
	Unemployed	58 (27.9)	23 (31.1)	16 (26.7)	7 (50.0)	11 (22.9)	
Ethnicity							
	Caucasian	109 (89.3)	5 (87.8)	54 (90.0)	11 (78.6)	44 (91.7)	*p* = .42
	Indigenous	6 (4.9)	65 (6.8)	3 (5.0)	2 (14.3)	1 (2.1)	
	Other	7 (5.7)	4 (5.4)	3 (5.0)	1 (7.1)	3 (6.3)	
Location							
	Small rural location	31 (25.4)	19 (25.7)	15 (25.0)	4 (28.6)	12 (25.0)	*p* = .89
	Small to medium city (10,000—200,000)	36 (29.5)	19 (25.7)	15 (25.0)	4 (28.6)	17 (35.4)	
	Large city (over 200,000)	55 (45.1)	36 (48.6)	30 (50.0)	6 (42.9)	19 (39.6)	
Mental health characteristics							
	Taking psychotropic medications	62 (50.8)	45 (60.8)	41 (68.3)	4 (28.6)	17 (35.4)	*p* = .006
Years with alcohol problems							
	0–5 years	57 (46.7)	34 (45.9)	25 (41.7)	9 (64.3)	23 (47.9)	*p* = .20
	6–10 years	25 (20.5)	15 (20.3)	12 (20.0)	3 (21.4)	10 (20.8)	
	More than 10 years	40 (32.8)	25 (33.8)	23 (38.3)	2 (14.3)	15 (31.3)	
Previously received treatment							
	Yes	42 (34.4)	25 (33.8)	23 (38.3)	2 (14.3)	17 (35.4)	*p* = .09
	No	80 (65.6)	49 (66.2)	37 (61.7)	12 (85.7)	31 (64.6)	
Type of treatment received							
	Alcoholics anonymous	21 (17.2)	15 (20.3)	14 (23.3)	1 (7.1)	6 (12.5)	*p* = .18
	Individual/group psychotherapy or counselling	23 (18.9)	14 (18.9)	13 (21.7)	1 (7.1)	9 (18.8)	*p* = .21
	Medical treatment	7 (5.7)	5 (6.8)	5 (8.3)	0 (0.0)	2 (4.2)	*p* = .22

^a^Analyses compare guided and self-guided Preference Trial participants

^b^One non-binary individual was not included in the analysis

The out of SK province participants accepted into the self-guided ACC (n = 48) had demographics comparable to the preference trial groups: they were primarily in middle adulthood (M = 44.75, SD = 13.67, range = 20–67), women (62.5%), Caucasian (91.7%), etc. (see Table 1 for details).

### Primary outcomes

Table 2 presents the pre-treatment to follow-up means and standard deviations for the primary outcome variables.

**Table 2:** Pre-treatment to 3-month follow-up observed mean and standard deviations for primary outcomes

Variable	All patients (*n* = 122)	All Preference Trial patients (*n* = 74)	Preference Trial (*n* = 74)		Accepted for Self-Guided Only (*n* = 48)	Statistical^a^ Significance *χ*^2^ or *t*-test
			Guided (*n* = 60)	Self-Guided (*n* = 14)	Met Severity criteria but Out-of-Province (*n* = 48)	
TLFB						
Pretreatment	37.24 (17.38)	37.82 (18.30)	38.85 (17.70)	33.43 (20.79)	36.33 (16.02)	*p* = .32
Midtreatment	15.20 (15.42)	15.79 (16.51)	16.43 (17.74)	12.40 (6.60)	14.17 (13.47)	*p* = .48
Posttreatment	13.67 (15.06)	13.65 (14.12)	14.08 (15.02)	11.33 (7.94)	13.69 (16.64)	*p* = .60
Followup	16.58 (14.78)	17.00 (15.76)	16.61 (16.37)	18.75 (13.44)	15.97 (13.46)	*p* = .73
HDD						
Pretreatment	4.11 (1.98)	3.99 (2.00)	4.13 (1.96)	3.36 (2.10)	4.31 (1.97)	*p* = .19
Midtreatment	1.65 (2.02)	1.62 (1.93)	1.75 (2.04)	0.90 (0.99)	1.69 (2.20)	*p* = .20
Posttreatment	1.36 (2.02)	1.37 (1.95)	1.51 (2.03)	0.67 (1.41)	1.35 (2.15)	*p* = .24
Followup	1.70 (2.06)	1.70 (1.97)	1.58 (1.98)	2.25 (1.98)	1.70 (2.22)	*p* = .39

*TLFB* Time Line Follow Back, *HDD* Heavy Drinking Days

^a^Analyses compare guided and self-guided Preference Trial participants

The mixed model analysis revealed a significant *time* effect (β =  − 2.64, SE 0.66; p < 0.001) on the decrease of TLFB scores over time, with a large pre-to-post Cohen’s effect size of d = 0.97 (95% CI [0.49, 1.45]). However, there was no significant effect (p = 0.28) of *group* (therapist- versus self-guided) or the *group* × *time* interaction (p = 0.34). *Time* remained significant (p < 0.001) after controlling for other clinically relevant variables (i.e., psychotropic medication use, lessons completed, expectancy and credibility, and readiness to change).

Similarly, the mixed model analysis showed a significant *time* effect (β =  − 0.34, SE 0.07; p < 0.001) on the decrease of HDD scores over time, with a large pre-to-post Cohen’s effect size of d = 1.19 (95% CI [0.69, 1.68]). There was no significant effect of *group* (p = 0.06) or *group* × *time* interaction (p = 0.06). *Time* remained significant (p < 0.001) after controlling for psychotropic medication use, lessons completed, expectancy and credibility, and readiness to change. Further, there was a sustained decrease from pre-treatment to follow-up on both the TLFB (d = 0.70, 95% CI [0.23, 1.17]) and HDD (d = 0.96, 95% CI [0.48, 1.45]).

### Secondary outcomes

See Table 3 for the pre-treatment to follow-up means and standard deviations of the secondary outcome variables.

**Table 3:** Pre-treatment to 3-month follow-up observed mean and standard deviations for secondary outcomes

Variable	All patients (*n* = 122)	All Preference Trial patients (*n* = 74)	Preference Trial (*n* = 74)		Accepted for Self-Guided Only (*n* = 48)	Statistical^a^ Significance *χ*^2^ or *t*-test
			Guided (*n* = 60)	Self-Guided (*n* = 14)	Met Severity criteria but Out-of-Province (*n* = 48)	
AUDIT						
Pretreatment	22.65 (5.76)	23.11 (6.19)	24.07 (6.01)	19.00 (5.39)	21.94 (5.00)	*p* = .005
Posttreatment	16.42 (8.09)	18.00 (8.41)	18.44 (8.89)	15.86 (5.49)	13.93 (6.97)	*p* = .48
Followup	14.44 (7.59)	14.80 (8.43)	15.11 (8.69)	13.38 (7.48)	13.90 (6.22)	*p* = .60
PHQ-9						
Pretreatment	10.71 (5.71)	11.35 (5.85)	11.98 (5.69)	8.64 (5.98)	9.73 (5.39)	*p* = .05
Midtreatment	6.76 (5.09)	6.75 (5.13)	6.74 (4.61)	6.80 (7.67)	6.78 (5.08)	*p* = .97
Posttreatment	6.13 (5.35)	6.25 (5.54)	6.50 (5.53)	4.89 (5.73)	5.94 (5.12)	*p* = .43
Followup	5.55 (4.73)	5.89 (4.79)	5.67 (4.73)	6.88 (5.22)	5.03 (4.69)	*p* = .53
GAD-7						
Pretreatment	8.90 (5.45)	9.27 (5.52)	10.08 (5.36)	5.79 (4.95)	8.33 (5.35)	*p* = .008
Posttreatment	4.93 (4.44)	5.28 (4.37)	5.55 (4.70)	4.00 (1.85)	4.40 (4.57)	*p* = .37
Followup	4.84 (4.48)	4.84 (3.86)	5.06 (4.06)	3.88 (2.75)	4.83 (5.37)	*p* = .44
PACS						
Pretreatment	17.02 (5.95)	17.24 (6.12)	17.60 (6.06)	15.71 (6.37)	16.69 (5.72)	*p* = .30
Posttreatment	12.27 (6.43)	12.76 (5.80)	12.95 (6.12)	11.86 (4.09)	11.53 (7.33)	*p* = .64
Followup	10.67 (5.91)	11.25 (6.28)	10.67 (5.88)	13.88 (7.72)	9.79 (5.28)	*p* = .20
CEQ						
Midtreatment	22.18 (4.02)	22.10 (4.19)	22.32 (4.02)	20.90 (5.09)	22.33 (3.75)	*p* = .33

*AUDIT* Alcohol Use Disorder Identification Test, *PHQ-9* Patient Health Questionnaire-9 item, *GAD-7* General Anxiety Disorder-7 item, *PACS* Penn Alcohol Craving Scale, *CEQ* Credibility and Expectancy Questionnaire

^a^Analyses compare guided and self-guided Preference Trial participants

The mixed model analyses conducted for the secondary outcome variables showed that there was a significant effect of *time* for patients’ scores on the AUDIT (β =  − 1.32, SE 0.28; p < 0.001), PACS (β =  − 1.28, SE 0.28; p < 0.001), BSCQ (β = 19.48, SE 6.68; p = 0.004), GAD-7 (β =  − 0.74, SE 0.18; p < 0.001), PHQ-9 (β =  − 0.88, SE 0.19; p < 0.001), and SDS (β =  − 1.13, SE 0.30; p < 0.001). There was no significant effect of *group* or *group* × *time* interaction for any of the secondary outcome variables: AUDIT (group: p = 0.10; interaction: p = 0.37), PACS (group: p = 0.31; interaction: p = 0.14), BSCQ (group: p = 0.14; interaction: p = 0.15), GAD-7 (group: p = 0.12; interaction: p = 0.17), PHQ-9 (group: p = 0.07; interaction: p = 0.18), or SDS (group: p = 0.32; interaction: p = 0.71). *Time* also remained significant (p < 0.001) for all secondary outcomes after controlling for the effects of other clinically relevant variables (i.e., psychotropic medication use, lessons completed, expectancy and credibility, and readiness to change).

### Additional outcomes

#### Treatment satisfaction and engagement

Preference trial patients completed, on average, 6.39 (SD = 2.32) lessons, which was not significantly different (p = 0.34) between those in the therapist- versus self-guided ACC. Based on their first and last log-in to the online treatment platform, therapist-guided patients spent more days (M = 50.58, SD = 24.09) on the course than self-guided patients (M = 36.71, SD = 31.24), but the mean group difference was not statistically significant (p = 0.07). Further, therapist-guided patients (M = 22.04, SD = 13.70) had significantly higher course log-ins (p = 0.04) compared to self-guided patients (M = 13.69, SD = 7.79).

Of the preference trial patients who completed post-treatment satisfaction questionnaires (n = 45), most reported satisfaction with the course overall (88.9%), satisfaction with the course materials (91.1%), increased confidence in managing their symptoms (86.7%), and increased motivation to seek other treatment if needed (91.1%). No significant differences in engagement and satisfaction were found between therapist- and self-guided patients (p range: 0.08–0.94), apart from satisfaction with course materials; significantly (p = 0.002) more patients in the therapist-guided ACC (97.3%) reported satisfaction with the course materials than patients in the self-guided ACC (62.5%), with therapist-guided patients being 1.56 times more likely to be satisfied with course materials compared to self-guided patients. See Table 4 for details on patients’ treatment engagement and satisfaction.

**Table 4:** Treatment engagement and satisfaction by group

Variable	All participants (*n* = 75)	All Preference Trial participants (*n* = 45)	Preference Trial (*n* = 45)		Accepted for Self-Guided (*n* = 30)	Statistical Significance^a^ *χ*^2^ or *t*-test
			Guided (*n* = 37)	Self-Guided (*n* = 8)	Out-of-Province Met Severity Criteria (*n* = 30)	
	*n* (%)	*n* (%)	*n* (%)	*n* (%)	*n* (%)	
Treatment ratings						
Satisfied overall	67 (89.3)	40 (88.9)	34 (91.9)	6 (75.0)	27 (90.0)	*p* = .17
Satisfied with materials	70 (93.3)	41 (91.1)	36 (97.3)	5 (62.5)	29 (96.7)	*p* = .002
Increased confidence managing symptoms	66 (88.0)	39 (86.7)	32 (86.5)	7 (87.5)	27 (90.0)	*p* = .94
Increased motivation seeking other treatment	67 (89.3)	41 (91.1)	35 (94.6)	6 (75.0)	26 (86.7)	*p* = .08
Course was worth the time	74 (98.7)	45 (100.0)	37 (100.0)	8 (100.0)	29 (96.7)	^b^
Would recommend the course to a friend	75 (100.0)	45 (100.0)	37 (100.0)	8 (100.0)	30 (100.0)	^b^

Engagement	(*n* = 122)	(*n* = 74)	(*n* = 60)	(*n* = 14)	(*n* = 48)	
Completed majority of lessons (i.e. 5 or more)	88 (72.1)	56 (75.7)	47 (78.3)	9 (64.3)	32 (66.7)	*p* = .27
Completed all lessons	74 (60.7)	46 (62.2)	38 (63.3)	8 (57.1)	28 (58.3)	*p* = .67
Lessons completed M (SD)	6.21 (2.46)	6.39 (2.32)	6.52 (2.20)	5.86 (2.80)	5.94 (2.67)	*p* = .34
Days between first and last log-in M(SD)	46.48 (24.05)	47.96 (25.94)	50.58 (24.09)	36.71 (31.24)	44.21 (20.85)	*p* = .07

^a^Analyses refer to comparison of guided and self-guided Preference Trial participants

^b^Unable to perform analysis due to no difference between the two groups

#### Negative effects

Of the preference trial patients who completed the post-treatment negative effects questions (n = 45), a majority (n = 41; 91.1%) did not report experiencing negative effects. Three patients in the therapist-guided ACC and one patient in the self-guided ACC reported experiencing some negative effects during treatment. Negative effects experienced by therapist-guided patients included: temporary increases in depressive symptoms (n = 1), losing one’s job after disclosing alcohol difficulties to their boss (n = 1), and an alcohol withdrawal-like effect (i.e., increased craving for sugar; n = 1). One self-guided patient reported being “very shaken for about a week” with “some visual disturbance for three days” and experiencing “poor quality sleep.”

### Exploratory analyses

Separate exploratory analyses were conducted to assess if the ACC was associated with reduced TLFB and HDD scores among patients residing outside of SK who were only eligible for the self-guided ACC (n = 48). The results of the mixed model analysis predicting TLFB scores over time showed that there was a significant effect of *time* (β =  − 2.03, SE 0.62; p = 0.001), with a large pre-to-post Cohen’s effect size of d = 1.32 (95% CI [0.88, 1.76]). Likewise, the mixed model analysis predicting HDD scores over time revealed a significant decrease (β =  − 0.31, SE 0.09; p < 0.001), with a large pre-to-post Cohen’s effect size of d = 1.04 (95% CI [0.61, 1.46]) on HDD. Furthermore, there was a sustained large effect from pre-treatment to follow-up on both the TLFB (d = 0.99, 95% CI [0.57, 1.41]) and HDD (d = 0.92, 95% CI [0.50, 1.34]). This self-guided group of patients residing outside of SK who were only eligible for the self-guided ACC was also comparable to the two preference trial groups in terms of treatment engagement and satisfaction (see Table 4).

## Discussion

The current preference trial aimed to elucidate how patients’ preferences for therapist- versus self-guided ICBT may inform the implementation of ICBT for alcohol misuse in routine care. Specifically, we examined (a) whether patients preferred therapist- versus self-guided ICBT, (b) if there were differences between those who preferred therapist- versus self-guidance, and (c) if patients who selected their preferred approach, therapist- or self-guided ICBT, differed in their outcomes, treatment engagement, and treatment satisfaction. As a natural comparison, the current study explored if ICBT for alcohol misuse was associated with reduced drinking and comorbid mental health difficulties, and treatment engagement and satisfaction among patients who resided outside of SK and, therefore, were only eligible for self-guided ICBT.

Of note, while past research shows that therapist- and self-guided ICBT can produce similar effects [10–13], 81.1% of the preference trial patients chose therapist-guided ICBT. These patients were more likely to be taking psychotropic medications, as well as to report higher drinking difficulties and anxiety symptoms compared to those who chose self-guided ICBT, thus suggesting that patients appear to appropriately self-select themselves into therapist-guided ICBT. Understanding the demand for therapist-guided ICBT is an important consideration when offering ICBT, as past research shows that patients’ preferences can impact enrollment [15, 16]. In line with our predictions informed by past research [7, 9, 10, 12, 13], our primary results showed that therapist- and self-guided ICBT were associated with reduced drinking following ICBT. Further, consistent with previous studies (e.g., [10–12]) and in contrast with other studies (e.g., [9, 17]), there was no significant effect of patients’ preference for therapist- or self-guided ICBT on their drinking outcomes over time. As noted earlier, there are no studies that can be benchmarked with our study in terms of preference for self- or therapist-guidance. However, when benchmarked for self- or therapist-guided ICBT for alcohol misuse, the effect sizes obtained from the current study for the primary outcomes are comparable to previous studies (e.g., [12]).

Interestingly, after completing ICBT, there was a significant concomitant improvement in AUDIT scores, alcohol craving, and confidence with controlling alcohol cravings, as well as in anxiety, depression, and overall functional impairment. Although such findings concur with previous studies (e.g., [11, 12]), it is unclear how the ACC influenced reduction in comorbid mental health difficulties, given the small sample in the current study. However, it is possible that patients’ comorbid mental health difficulties were alleviated via their increased confidence with controlling alcohol cravings, along with their absorption and implementation of the psychoeducational information and cognitive-behavioural techniques outlined in the ACC (see [40]). Further studies with larger samples are needed to illuminate the potential mechanisms of change in comorbid mental health difficulties with ICBT for alcohol misuse.

Similarly, in terms of treatment engagement and satisfaction, the majority of preference trial patients completed five or more lessons, reported overall treatment satisfaction, increased confidence in managing their symptoms, and increased motivation to seek other treatment if needed. Further, almost all preference trial patients indicated that the ACC was worth their time and that they were willing to recommend the ACC to their friends. Therapist- and self-guided patients did not differ in any measure of treatment engagement or satisfaction except that more therapist-guided patients reported satisfaction with the course materials compared to self-guided patients.

Further, additional exploratory analyses of the exclusively self-guided patients residing outside of SK revealed a decrease in their drinking outcomes over time. This finding provides support for the association of self-guided ACC with improved drinking outcomes among patients with alcohol misuse difficulties across Canada. Given that few resources are needed to provide self-guided ICBT, offering ICBT to this group longer term is feasible.

The promising results from the current preference trial may reflect the benefits of patients choosing their guidance level in ICBT for alcohol misuse, as it is unclear if we would have seen the same results if the preference trial patients preferring therapist guidance were imposed self-guided ICBT, or vice versa. Still, our finding that patients in the exclusively self-guided group of patients residing outside of SK also exhibited significantly improved outcomes suggests that self-guided ICBT has a potential to be scaled up across Canada and beyond as an accessible Internet-delivered treatment option.

## Limitations and future directions

There are various limitations to the current study. First, the sample size of preference trial patients enrolled in self-guided ICBT was small, indicating that most preference trial patients preferred therapist-guided ICBT. Although this was based on patients’ preferences, and repeated-measures mixed-model analyses can produce accurate inferences with small samples (see [41, 42]), our findings comparing therapist- versus self-guided ICBT should be viewed with caution in terms of reproducibility and generalizability. Nonetheless, our results are consistent with both previous research (e.g., [10, 11]) and the current exploratory analysis of patients outside of SK who were only eligible for self-guided ICBT.

Second, although the groups were comparable demographically and the potential effects of clinically relevant variables were controlled for in the final analyses, we did not include a control group in this study as this was not the study focus and has been subject of many past trials (e.g., see [7]). As such, without a control group, this study does not address how ICBT outcomes compare to a control group. We acknowledge that all improvements in outcomes observed in this study could have been a result of assessment reactivity, natural improvements over time, patient desire to report positive outcomes to the investigator, or some other co-occurring treatment and not necessarily due to ICBT. Further, in our exploratory analyses of residents outside of SK, we did not ask patients about their preferences for therapist- or self-guided ICBT, which would have been interesting to examine if patients indicate a preference for therapist-guided ICBT when it is not available. In the future, it would be valuable to further explore if, in addition to their experience of guidance-related preferences, patients have other preferences regarding ICBT for alcohol misuse (e.g., mode of contact, timeline of ICBT, and comorbid treatment resources). Moreover, beyond examining the impact of preferences on treatment engagement, satisfaction, and outcomes, it would be beneficial to explore how such preferences impact enrollment in ICBT.

## Conclusions

The current study shows that, while most patients prefer therapist-guidance, ICBT for alcohol misuse was associated with reduced alcohol use and comorbid mental health difficulties over time irrespective of whether patients completed the ACC on their own or with therapist guidance in accordance with their preferences. Overall, patients preferring therapist- or self-guided ICBT also did not differ in their treatment engagement or satisfaction, even though the patients choosing therapist-guided ICBT were more likely to report psychotropic medication use, higher drinking difficulties, and greater anxiety symptoms compared to those in self-guided ICBT. Further, the congruent and promising outcomes seen among exclusively self-guided patients (who were not eligible for therapist-guidance) across Canada provide compelling evidence to support the use of ACC as a self-guided and Internet-delivered treatment to reduce alcohol misuse. If replicated in a larger sample, the ACC could be scaled up broadly throughout the country.

## Data Availability

All data used and reported in the current study are available from the corresponding author upon reasonable request.

## Abbreviations

ACC: Alcohol change course
AUDIT: Alcohol use disorder identification test
BSCQ: Brief situational confidence questionnaire
CEQ: Credibility and expectancy questionnaire
GAD-7: Generalized anxiety disorder-7
HDD: Heavy drinking days
ICBT: Internet-delivered cognitive behaviour therapy
OTU: Online therapy unit
PACS: Penn alcohol craving scale
PHQ-9: Patient health questionnaire-9
RCQ-TV: Readiness to change questionnaire treatment version
SDS: Sheehan disability scale
SK: Saskatchewan
TLFB: Timeline follow-back
